# Overweight in Older Adults: A Follow-Up of Fifteen Years of the SABE Survey

**DOI:** 10.3390/ijerph20065098

**Published:** 2023-03-14

**Authors:** Tânia Aparecida de Araujo, Isabela Martins Oliveira, Tarsila Guimarães Vieira da Silva, Vanderlei Carneiro da Silva, Yeda Aparecida de Oliveira Duarte

**Affiliations:** 1 Leônidas and Maria Deane Institute, Fiocruz Amazônia, Manaus 69057-070, Brazil; 2 Faculty of Public Health, University of São Paulo, São Paulo 01246-904, Brazil

**Keywords:** nutritional status, overweight, obesity, aging, longitudinal study

## Abstract

Despite extensive research on overweight and obesity, there are few studies that present longitudinal statistical analyses among non-institutionalized older adults, particularly in low- and middle-income countries. This study aimed to assess the prevalence and factors associated with excess weight in older adults from the same cohort over a period of fifteen years. A total of 264 subjects aged (≥60 years) from the SABE survey (Health, Wellbeing and Aging) in the years 2000, 2006, 2010, and 2015 in the city of São Paulo, Brazil, were evaluated. Overweight was assessed by a BMI of ≥28 kg/m^2^. Multinomial logistic regression models adjusted for sociodemographic and health data were used to assess factors associated with excess weight. After normal weight, overweight was the most prevalent nutritional status in all evaluated periods: 34.02% in 2000 (95%CI: 28.29–40.26); 34.86% in 2006 (95%CI: 28.77–41.49%); 41.38% in 2010 (95%CI: 35.25–47.79); 33.75% in 2015 (95%CI: 28.02–40.01). Being male was negatively associated with being overweight in all years (OR: 0.34 in 2000; OR: 0.36 in 2006; OR: 0.27 in 2010; and OR: 0.43 in 2015). A greater number of chronic diseases and worse functionality were the main factors associated with overweight, regardless of gender, age, marital status, education, physical activity, and alcohol or tobacco consumption. Older adults with overweight and obesity, a greater number of chronic diseases, and difficulties in carrying out daily tasks required a greater commitment to healthcare. Health services must be prepared to accommodate this rapidly growing population in low- and middle-income countries.

## 1. Introduction

Aging populations are poised to become the next global public health challenge [1]. It is estimated that by 2050, one in five people worldwide will be 60 years old or older, totaling 2 billion people. [2]. In Brazil, older adults represent the fastest growing age group [3]. It is uncertain, however, whether increased life expectancy is also associated with a better health status [4].

It is known that in addition to socioeconomic changes, aging is accompanied by progressive physiological changes that can impact functionality, autonomy, and nutritional status [5]. Weight loss can be caused by various factors such as changes in smell and taste [6], as well as reductions in lean mass, bone tissue, and muscle strength [7]. Additionally, changes in body composition are related to increased body weight, such as re-distribution of and increase in fat mass (especially in the abdominal region) and reductions in lean mass [8], which may lead to decreased energy expenditure rates.

There is still much debate about the optimal weight in old age. The modified BMI classifications [9] suggest that a higher body weight is expected among older adults. Although cross-sectional studies have shown an increase in body weight in this population [10,11], the most common recommendation among specialists is the prevention of low weight [9]. Thus, while the world is facing alarming rates of overweight and obesity (especially among young people and adults), overweight does not seem to be a major concern among older adults.

Longitudinal studies conducted in developed countries [12,13] have shown an increase in overweight, obesity, and abdominal obesity even among older adults. However, in developing countries, there is a scarcity of results from longitudinal studies with this population. For example, in an extensive systematic review of longitudinal studies conducted from 2012 to 2020 that identified the association between physical activity and the onset of obesity, only one study from a developing country, Mexico, was identified among the fourteen countries evaluated [14].

Kawai et al. [15] draw attention to the immense difficulty of conducting longitudinal studies, especially among older adults. According to the authors, in addition to the higher mortality in this population, physical limitations, low education, and economic situation, for example, make it difficult for older adults to participate in these studies. In developing countries, where investment in research is very limited, the difficulties in carrying out these studies can be even greater.

As developing countries, including those in Latin America, experience rapid population aging, they face significant challenges in providing adequate healthcare for their growing older adult populations. Conducting longitudinal studies in these locations could help to shed light on the unique aging patterns in these populations, which could in turn provide valuable insights for improving the health and well-being of older adults in these regions.

The objective of this study is to evaluate the prevalence and factors associated with excess weight in older adults, followed in a fifteen-year follow-up.

## 2. Materials and Methods

### 2.1. Sample and Study Population

The SABE study—Health, Well-Being and Aging—began in 2000 as a population-based survey to assess the living conditions and health of older adults residing in seven urban centers in Latin America and the Caribbean. In Brazil, the center chosen was the city of São Paulo, which, under the coordination of members of the Epidemiology department of the School of Public Health at the University of São Paulo, became a multi-cohort longitudinal study. Thus, the older adults assessed in 2000 were revisited in 2006, 2010, and 2015, comprising cohort A.

This study used panel data from the SABE study spanning a 15-year period (2000 to 2015). The sample for the SABE study was selected using a two-stage cluster sampling method, with proportional allocation based on size, at the census sector and household levels. In the first wave of data collection (cohort A—2000), due to the low population density, the sample sizes for the 75 years and older age group were increased. Additionally, the male samples were adjusted to match the female samples to account for the higher mortality rate in the male population. For the expanded groups, the sample composition was determined freely [16].

For this study, data were used only from individuals from cohort A, which started in 2000 and of which participants remained in the study until the last wave of collections in 2015 (n = 356). Those who did not have complete anthropometric data were excluded (n = 92), making a final sample of n = 264.

Trained interviewers collected data in household interviews, using a structured questionnaire that addressed socioeconomic variables, general health status, living conditions, and anthropometric measurements. In addition, to control the quality of the interviews and reduce information bias, the questionnaires returned by the interviewers went through a critical analysis of completion and initial consistency. Details about the methodology are described in another publication [17].

### 2.2. Variables

#### 2.2.1. Dependent Variable

The dependent variable was overweight (≥28 kg/m^2^) assessed by BMI (kg/m^2^). To calculate BMI, body mass (in kilograms) was divided by the square of height (in meters). Body mass was measured using a calibrated portable scale (SECA^®^ brand) with a capacity of 150 kg and sensitivity of ½ kg, and height was measured using a stadiometer fixed to a 2 m wall with a sensitivity of 1 mm. All anthropometric variables were measured in triplicate and the mean value was used for the analyses, according to the Frisancho [18] standardization.

For the classification of BMI, we used the cutoff points adopted by the Pan American Health Organization (PAHO) for the SABE [19] study: normal weight (>23 and <28 kg/m^2^), underweight (≤23 kg/m^2^), and overweight (≥28 kg/m^2^).

#### 2.2.2. Independent Variables

Sociodemographic characteristics included gender, age (both continuous and categorized as 60–70 years, 71–80 years, and >80 years), years of education (categorized as illiterate, 1–6 years, and ≥7 years), and marital status (categorized as married, widowed, and single/divorced).

Clinical characteristics: The respondents were asked whether a doctor or nurse had ever informed them of the diagnosis of any of the following diseases: hypertension, diabetes, cardiovascular diseases, and arthropathies. Number of chronic diseases was categorized as 0 or 1 and ≥2 diseases, and self-assessment of health was categorized as very good/good and regular/bad/very bad.

Functional characteristics: The Katz scale [20] was used to assess the difficulty in performing ≥1 basic activity of daily living (ADL) and ≥1 instrumental activity of daily living (IADL). The ADLs considered were getting dressed alone, crossing a room, eating, bathing, using the bathroom, and getting up from a bed or chair. The IADLs considered were preparing meals, managing one’s own money, using the telephone, using transportation, taking medications, shopping, and performing light and heavy household chores.

Behavioral characteristics: We assessed smoking status (never smoked, ever smoked and no longer smokes, and currently smokes); alcohol consumption (we asked how many days per week the older adults had consumed some alcoholic beverage and categorized as low consumption (does not consume or <1×/week), moderate (1 to 3×/week), and high (4×/week to every day)) [21]; and for physical activity, we asked whether the respondent performed physical activity and how many days per week (categorized as yes (≥3×/week) and no (<3×/week)).

Anthropometric characteristics included waist circumference (WC) and calf circumference (CC). WC was measured using an inelastic tape measure at the midpoint between the last rib and the iliac crest, with the abdomen relaxed at the end of expiration and the area measured free of clothing. If it was not possible to measure the midpoint, the "natural waist" measurement was used, also with a relaxed abdomen. CC was measured using an inelastic tape measure at the midpoint of the dominant leg (as indicated by the older adults) with the older adult in a sitting position, forming a 90º angle with the knee, following the standardization of Lohman et al. [22].

### 2.3. Statistical Analysis

Considering the complex research design, survey weights were used to estimate the prevalence of overweight and other nutritional statuses (underweight and normal weight).

To ensure the reliability of the evaluated data, and considering the significant losses that occurred during the follow-up, we compared the “lost” subjects to the interviewees in terms of age, BMI, WC, and CC. As all of these variables were continuous, we checked their normality using the Shapiro–Wilk test and histograms. For those variables that met the normality criteria, we used Student’s t-test to compare means. For continuous variables without a normal distribution, we used the Wilcoxon test.

Categorical variables were analyzed using weighted proportions. Adjusted cross-sectional multinomial logistic regression models were used to estimate the odds ratios (ORs) and their 95% confidence intervals (CI) for the outcome of overweight with demographic factors (sex) and clinical factors, while controlling for age, education, marital status, alcohol consumption, smoking, and physical activity. Differences between β values were estimated using the Wald test, comparing overweight individuals with normal weight individuals as the reference category. Tests with a *p*-value < 0.05 were considered statistically significant. All analyses were performed using STATA 14.2.

The study was approved by the Ethics Committee of the Faculty of Public Health, University of São Paulo, under control numbers 315 (2000), 83 (2006), 2044 (2010), and 3,600,782 (2015). Participants were asked to read and sign a consent letter before the assessments and interviews began.

## 3. Results

**Figure 1:**
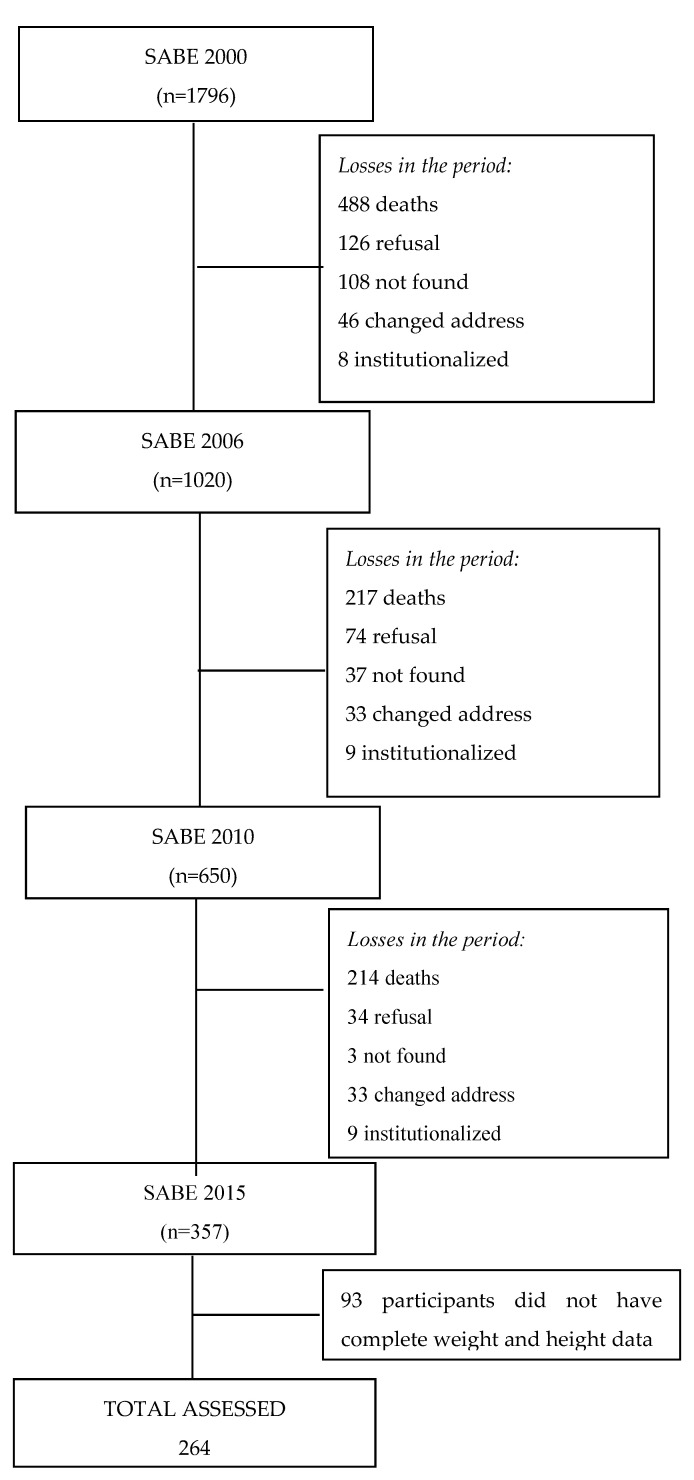
Sample of older adults from cohort A, with complete data on weight and height, in each data collection. SABE study, from 2000 to 2015.

The final sample of this study consisted of 264 participants who were assessed in 2000, 2006, 2010, and 2015. During the evaluation period, 65.3% of these losses occurred due to deaths and 34.7% due to other reasons such as institutionalization, refusals, change of address, and being unable to locate the participants. The flow chart of losses is presented in Figure 1.

The data for individuals lost due to death or other reasons are presented in Table 1. In comparison to the evaluated older adults, those who died during the study follow-up had lower BMI and CC values. However, participants who were lost due to other reasons did not show differences in terms of age or anthropometric values from those who were assessed.

Descriptive data from the baseline (year 2000) are presented according to nutritional status in Table 2. Since this is a sample of individuals who were followed for fifteen years from 2000, the majority of them were under 70 years of age at baseline. In the same year, however, almost half of the sample had already been diagnosed with hypertension. Among hypertensive individuals, overweight was more prevalent among women. Older adults classified as having a normal weight reported engaging in more physical activity (more than 3 times a week).

**Figure 2:**
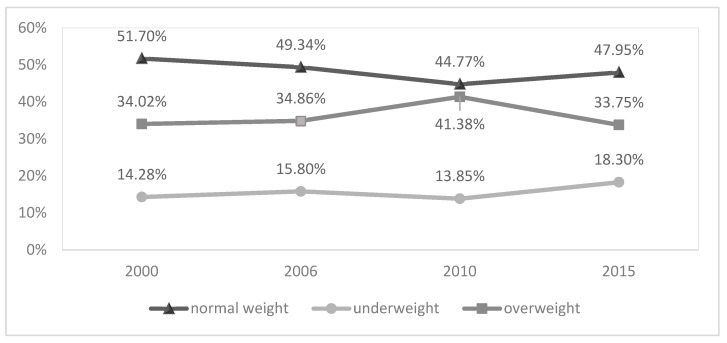
Nutritional status of older adults from the SABE study, first wave (2000), second wave (2006), third wave (2010), and fourth wave (2015). São Paulo, 2000 to 2015.

The weighted prevalence of underweight, normal weight, and overweight were compared in 2000, 2006, 2010, and 2015. Except for the fourth wave in 2015, the prevalence of overweight followed an increasing trend in all evaluated waves, reaching its lowest percentage difference between normal weight and overweight in 2010. See Figure 2.

The multinomial logistic regression model yielded raw and adjusted values of factors associated with excess weight in each wave of the study. The results showed that, regardless of age, education, marital status, alcohol consumption, smoking, and physical activity, men had a lower risk of being overweight compared to women (OR: 0.34–2000; OR: 0.36–2006; OR: 0.27–2010; and OR: 0.43–2015). On the other hand, for the entire sample, factors that increased the risk of being overweight were worse functionality (greater difficulty in performing ADLs and IADLs), diagnosis of hypertension, arthropathies, or having two or more non-communicable chronic diseases together. Table 3 shows the details.

## 4. Discussion

Overweight and obesity are known to predispose individuals to disability and reduced physical functioning [8]. This study, which monitored the same population of older adults over a period of fifteen years, found an increase in the prevalence of overweight and obesity in the first three follow-up waves. Only in the last wave, with an average age of 77 among those evaluated, did the prevalence of overweight decrease.

Previous longitudinal studies indicate an increase in BMI up to the age of 65 [23], stabilizing and starting to decrease after the age of 80 [24]. In a recent 15-year follow-up study, the authors reported that body weight stabilized up to the age of 65 and started to decrease after the age of 66 [25]. In another study carried out with the first three waves of the SABE study, which had a 10-year follow-up, a decrease in body weight was observed after the age of 70 [26].

The global prevalence of overweight and obesity appears to be higher among women than men [23], especially after the age of 50 [27]. The results found in this study confirm these findings, as men were less likely to be overweight than women in all evaluated waves. These results may be due to biological differences, such as the effect of menopause and sex hormones, for example, or to cultural and social factors that result in lower levels of education and a more sedentary lifestyle among women [27].

The increasing trend in the prevalence of overweight and obesity may be associated with economic development, urbanization, and the globalization of food systems [23,27]. These factors stimulate the production and availability of more processed and ultra-processed foods [28], which promote excessive consumption of energy-rich and nutrient-poor foods [27]. These types of foods may be particularly attractive to older adults, who often face challenges in purchasing and preparing food [29]. Furthermore, the modernization of lifestyles results in reduced physical activity, further contributing to the increase in body weight [26].

On the other hand, although children, adolescents, adults, and older adults may share some factors that contribute to overweight, the effects of overweight and obesity in old age are still uncertain [30]. For example, a meta-analysis that assessed mortality risks in older adults (aged 65 years or older) demonstrated that the increased risk of mortality occurred for individuals with a BMI <23 kg/m^2^ [31], while being overweight was not associated with an increased risk of mortality for older populations [32]. Contradicting these results, another study which included nearly 900,000 adults by the Prospective Studies Collaboration found a 30% increase in mortality risk for every 5-unit increase in BMI above 22.5–25 kg/m^2^ [33]. Results from this work demonstrated that the mean BMI of those participants who died during the study was also lower than those who were evaluated. According to Cetin [30], it is possible that individuals vulnerable to the negative impacts of obesity have a lower life expectancy, as weight gain is not necessarily associated with increased survival. In contrast, those who survive into old age may have greater resistance to the harmful effects of obesity.

Even though the risks of overweight and obesity among older adults are still divergent in the literature, our data revealed that those with overweight and obesity had a greater number of chronic non-communicable diseases and greater difficulty in basic and instrumental activities of daily living. This fact, per se, would indicate a worse health status.

Overweight and obesity are among the main preventable risk factors for diseases such as type 2 diabetes mellitus, fatty liver disease, hypertension, myocardial infarction, stroke, dementia, osteoarthritis, obstructive sleep apnea, and various types of cancer [33]. These conditions also predispose individuals to greater disability and decreased physical functioning. Data from the Health, Aging and Body Composition study, for example, revealed that adults and older adults classified as overweight or obese had a hazard ratio of 2.38 for incident disability over a 7-year follow-up period [34]. According to Silva [35], excess weight is one of the factors associated with declines in strength, mobility, and flexibility in older adults, thereby altering their ability to perform daily activities [36]. In 2000, being overweight was also associated with a worse self-assessment of health. Although this association is not well explored in the literature, Borim [37] reported that the prevalence of good/excellent self-assessment of health was significantly lower in older individuals with BMI ≥ 30 kg/m^2^ in the city of Campinas, Brazil. A greater number of associated diseases and disabilities may explain this association.

Batsis [8] states that although there are challenges in the diagnostic accuracy of obesity, regardless of the body composition or anthropometric measurement used, in older adults living in the community, obesity is associated with a worse prognosis for physical function. According to data from the Cardiovascular Health Study 1989–2015, an otherwise healthy lifestyle, including physical activity, diet, and weight control, can compress the number of years of disability.

The strengths of this study include long-term monitoring of the community’s older adults for a period of 15 years. The complexity of the sampling process and careful execution of the research adds to its important internal validity. Weight and height values were directly measured rather than self-reported, increasing confidence in the findings. Additionally, using specific BMI cut-off points helped avoid overestimation of the prevalence of overweight and obesity. As some authors have noted, the WHO healthy weight range for adults may not be appropriate for older adults [31].

This manuscript also has some important limitations that are worth mentioning. Firstly, the data were analyzed cross-sectionally, which makes it difficult to establish causal relationships between the variables. However, the consideration of sample weight and adjusted regression models supports the reliability of the demonstrated results. Secondly, changes in weight and nutritional status may have occurred outside the study collection periods. Despite rigorous quality control, the diagnostic data for chronic diseases were obtained through self-reported accounts from the participants. Lastly, in the final wave of the study in 2015, a significant portion of those assessed (24.2%) were excluded from the anthropometric measurement as they were unable to stand on their feet due to physical limitations. Additionally, nearly half of the population (48.2%) was over 80 years old.

## 5. Conclusions

“While the clinical approach to overweight and obesity in older adults is still controversial, prevention remains a safe measure. Knowledge of the evolution of nutritional status in older adults, allowed by longitudinal follow-ups like this study, can be useful for decision-makers. Even at older ages, this study demonstrated a high prevalence of overweight, which is associated with worse health indicators such as the presence of chronic diseases and lower functionality. Including older adults in programs to prevent and treat overweight is a necessary measure that public health managers should consider.

Future research may indicate the effectiveness of measures such as diet, physical activity, and the promotion of healthy environments in reducing the prevalence of overweight and obesity in old age.

## Figures and Tables

**Table 1:** Age and anthropometric characteristics (means and medians) of older adults according to follow-up status in the SABE study, São Paulo, from 2000 to 2015.

	2000–2006 ^§^			2006–2010			2010–2015		
	Deaths	Other Losses ^¥^	Evaluated	Deaths	Other Losses	Evaluated	Deaths	Other Losses	Evaluated
Age (years)	78.0 ***	70.0	67.0	82.0 ***	76.0	73.0	83.0 ***	77.0	77.0
BMI (kg/m^2^)	25.1 ***	26.6	26.8	23.8 ***	25.8	26.0	25.6 **	26.9	27.3
WC (cm)	95.0	93.0 *	95.0	89.0	89.6	90.7	91.2	91.9	93.3
CC (cm)	34.0 ***	36.0	36.0	33.5 ***	35.0	35.0	33.9 ***	36.1	35.8

Student’s t-test; Wilcoxon test (non-parametric distribution); BMI: Body Mass Index; WC: waist circumference; CC: calf circumference. ^§^ 2000–2006: loss accounted for in 2006, last assessment carried out in 2000; 2006–2010: loss accounted for in 2010, last assessment carried out in 2006; 2010–2015: loss accounted for in 2015, last assessment carried out in 2010; ^¥^ lost to follow-up due to other reasons: change of address, not located, institutionalized, refusal. * *p*-value < 0.05; ** *p*-value < 0.01; *** *p*-value < 0.001.

**Table 2:** Sociodemographic and clinical characteristics of older adults according to nutritional status at baseline. SABE study, São Paulo, 2000.

	All N = 264 (%)	Normal Weight n = 129 (%)	Underweight n = 39 (%)	Overweight n = 96 (%)
Sex				
Female	65.4	53.4	68.4	82.4
Male	34.6	46.6	31.6	17.6
Age (years)				
60 to 70	79.2	82.0	72.8	77.5
70 to 80	20.5	17.7	26.5	22.2
≥80 years old	0.3	0.3	0.3	0.3
Education				
Illiterate	12.3	11.3	13.7	13.3
1 to 6 years	66.8	67.3	69.6	64.7
≥7 years	20.9	21.4	16.7	22.0
Marital status				
Married	63.1	64.9	60.9	61.2
Widower	11.2	11.1	7.1	13.2
Not married ^†^	25.7	24.1	32.0	25.7
Alcohol (consumption) ^‡^				
Low	79.1	72.4	84.0	87.2
Moderate	15.7	18.6	13.7	12.3
High	5.2	9.0	2.2	0.50
Smoking				
Never	60.9	51.9	62.0	74.2
Already smoked ^§^	29.1	35.2	21.2	23.0
Smoke	10.0	12.9	16.8	2.8
Physical activity				
Yes	35.1	38.8	29.6	32.0
No	64.9	61.2	70.4	68.0
Self-assessment of health ^¶^				
Good	53.1	58.4	51.7	45.2
Bad	46.9	41.9	48.3	54.9
Hypertension				
No	52.6	59.6	60.1	38.4
Yes	47.4	40.4	39.9	61.1
Diabetes				
No	87.6	89.6	87.9	84.5
Yes	12.4	10.5	12.1	15.5
Cardiovascular diseases				
No	90.9	93.9	91.7	86.0
Yes	9.1	6.1	8.3	14.0
Arthropathies				
No	77.1	82.6	80.3	67.4
Yes	22.9	17.4	19.7	32.6
Number of diseases ^¥^				
0 or 1	68.4	80.2	76.3	47.3
≥2	31.6	19.8	23.7	52.8
Difficulty in ≥1 ADL ^£^				
No	87.1	94.4	93.6	73.4
Yes	12.9	5.6	6.4	26.6
Difficulty in ≥1 IADL ^±^				
No	83.7	90.6	81.9	73.9
Yes	16.3	9.5	18.1	26.1

^†^ Single or divorced; ^‡^ alcohol consumption: low—does not consume or <1×/week; moderate—1 to 3×/week; and high—4×/week or every day; ^§^ you have already smoked, do not smoke anymore; ^¶^ Good: very good and good; Bad: regular, bad, and very bad; ^¥^ Chronic diseases: hypertension, diabetes, cancer, cardiovascular diseases, lung diseases, embolism/stroke, or arthropathies. ^£^ ADL—basic activities of daily living: getting dressed, having a bath, getting up from bed or chair, using the bathroom, walking across a room, and eating alone. ^±^ IADL—instrumental activities of daily living: preparing meals, using transport, using the phone, performing light and heavy household tasks, shopping, managing their own money, and taking medications.

**Table 3:** Association between sex and clinical conditions with overweight among older adults, according to wave/year of assessment. SABE study, São Paulo, 2000 to 2015.

	2000		2006		2010		2015	
	OR (CI 95%) Crude	OR (CI 95%) Adjusted ^†^	OR (CI 95%) Crude	OR (CI 95%) Adjusted	OR (CI 95%) Crude	OR (CI 95%) Adjusted	OR (CI 95%) Crude	OR (CI 95%) Adjusted
Sex ^‡^								
Female	1.00	1.00	1.00	1.00	1.00	1.00	1.00	1.00
Male	0.24 *** (0.12–0.50)	0.34 ** (0.16–0.74)	0.33 ** (0.16–0.68)	0.36 * (0.15–0.86)	0.26 *** (0.13–0.49)	0.27 ** (0.13–0.59)	0.34 *** (0.17–0.66)	0.43 * (0.20–0.93)
Self-assessment of health ^§^								
Good	1.00	1.00	1.00	1.00	1.00	1.00	1.00	1.00
Bad	1.92 * (1.08–3.41)	2.25 * (1.16–4.38)	0.80 (0.44–1.45)	1.12 (0.56–2.27)	0.92 (0.52–1.63)	1.07 (0.58–2.00)	1.08 (0.60–1.94)	1.24 (0.64–2.39)
Hypertension								
No	1.00	1.00	1.00	1.00	1.00	1.00	1.00	1.00
Yes	2.31 *** (1.30–4.11)	2.36 ** (1.27–4.40)	1.22 (0.64–2.34)	1.53 (0.74–3.17)	2.22 * (1.14–4.33)	2.80 ** (1.33–5.91)	1.65 (0.84–3.27)	1.74 (0.84–3.59)
Diabetes								
No	1.00	1.00	1.00	1.00	1.00	1.00	1.00	1.00
Yes	1.58 (0.68–3.66)	1.38 (0.53–3.61)	0.92 (0.44–1.91)	0.81 (0.36–1.81)	1.82 (0.94–3.53)	1.85 (0.87–3.97)	1.66 (0.90–3.06)	1.42 (0.72–2.80)
Cardiovascular diseases								
No	1.00	1.00	1.00	1.00	1.00	1.00	1.00	1.00
Yes	2.49 (0.90–6.93)	3.64 * (1.19–11.15)	0.87 (0.42–1.83)	0.99 (0.45–2.20)	0.81 (0.42–1.54)	0.88 (0.44–1.77)	1.70 (0.91–3.17)	2.46* (1.16–5.19)
Arthropathies								
No	1.00	1.00	1.00	1.00	1.00	1.00	1.00	1.00
Yes	2.30 ** (1.20–4.41)	1.53 (0.75–3.11)	2.01 * (1.09–3.73)	1.68 (0.86–3.27)	1.63 (0.92–2.89)	1.25 (0.63–2.49)	2.52 ** (1.39–4.57)	2.05 * (1.04–4.05)
Number of diseases ^¶^								
0 or 1	1.00	1.00	1.00	1.00	1.00	1.00	1.00	1.00
≥2	4.52 *** (2.39–8.53)	3.57 *** (1.80–7.10)	1.74 (0.94–3.23)	1.81 (0.92–3.55)	2.00* (1.12–3.59)	2.29 * (1.16–4.49)	2.05 * (1.08–3.88)	2.22 * (1.07–4.56)
Difficulty in ≥1 ADL ^¥^								
No	1.00	1.00	1.00	1.00	1.00	1.00	1.00	1.00
Yes	6.11 *** (2.32–16.10)	5.16 ** (1.85–14.43)	2.04 * (1.01–4.09)	1.81 (0.83–3.98)	2.47 ** (1.31–4.67)	2.37 * (1.12–5.01)	1.98 * (1.07–3.64)	1.88 (0.90–3.93)
Difficulty in ≥1 IADL ^£^								
No	1.00	1.00	1.00	1.00	1.00	1.00	1.00	1.00
Yes	3.38 ** (1.51–7.55)	2.82 ** (1.19–6.71)	2.40 ** (1.31–4.41)	2.28 * (1.15–4.54)	2.38 ** (1.35–4.21)	2.40 ** (1.25–4.63)	0.95 (0.53–1.72)	0.65 (0.31–1.39)

Multinomial logistic regression; Wald’s test. Comparison of overweight individuals with normal weight individuals (reference category). OR: odds ratio; C95%: 95% confidence interval, ^†^ Adjusted by sex, age, schooling, marital status, alcohol consumption, smoking and physical activity. ^‡^ Adjusted for age, schooling, marital status, alcohol consumption, smoking and physical activity. ^§^ Good: very good and good; Bad: regular, bad, and very bad. ^¶^ Chronic diseases: hypertension, diabetes, cancer, cardiovascular diseases, lung diseases, embolism/stroke, or arthropathies. ^¥^ ADL—basic activities of daily living: getting dressed, having a bath, getting up from bed or chair, using the bathroom, walking across a room, and eating alone. ^£^ IADL—instrumental activities of daily living: preparing meals, using transport, using the phone, performing light and heavy household tasks, shopping, managing their own money, and taking medications. * *p* < 0.05; ** *p* < 0.01; *** *p* < 0.001.

## Data Availability

The datasets analyzed in the current study were used under license and are not publicly available due to the policies of the SABE survey.
